# DeepMILO: a deep learning approach to predict the impact of non-coding sequence variants on 3D chromatin structure

**DOI:** 10.1186/s13059-020-01987-4

**Published:** 2020-03-26

**Authors:** Tuan Trieu, Alexander Martinez-Fundichely, Ekta Khurana

**Affiliations:** 1 Meyer Cancer Center, Weill Cornell Medicine, New York, NY 10065 USA; 2 Department of Physiology and Biophysics, Weill Cornell Medicine, New York, NY 10065 USA; 3 Institute for Computational Biomedicine, Weill Cornell Medicine, New York, NY 10021 USA; 4 Caryl and Israel Englander Institute for Precision Medicine, New York Presbyterian Hospital-Weill Cornell Medicine, New York, NY 10065 USA

**Keywords:** 3D genome, Non-coding mutation, Cancer, BCL2, MYC, Deep learning

## Abstract

Non-coding variants have been shown to be related to disease by alteration of 3D genome structures. We propose a deep learning method, DeepMILO, to predict the effects of variants on CTCF/cohesin-mediated insulator loops. Application of DeepMILO on variants from whole-genome sequences of 1834 patients of twelve cancer types revealed 672 insulator loops disrupted in at least 10% of patients. Our results show mutations at loop anchors are associated with upregulation of the cancer driver genes *BCL2* and *MYC* in malignant lymphoma thus pointing to a possible new mechanism for their dysregulation via alteration of insulator loops.

## Background

The human genome is organized into three-dimensional (3D) hierarchical structures such as chromosomal compartments, topologically associated domains (TADs), and chromatin loops. Chromosome conformation capture techniques such as Hi-C [1] and ChIA-PET [2] can be used to identify these 3D structures. In particular, ChIA-PET assays capture chromatin interactions between loci mediated by a specific protein [2, 3]. Multiple studies have found that chromatin loops mediated by CTCF and cohesin (SMC1, SMC3, RAD21, and either STAG2 or STAG1) bound on both anchors at the loop ends isolate genes from active enhancers and their disruption can cause dysregulation of nearby genes [3–7]. These chromatin loops are called insulator loops. Mutations at anchors of such insulator loops may break or weaken loops and allow proto-oncogenes to interact with enhancers outside of the loops or to inhibit regulatory elements of tumor suppressors from interacting with their proper target genes [5]. Thus, methods to identify the mutations that are likely to disrupt the insulator loops are needed.

To the best of our knowledge, there is currently no method to identify mutations that can alter insulator loops. A natural approach is to model insulator loops and then observe how loops are changed in the presence of mutations. However, modeling insulator loops is challenging because the precise DNA sequence rules and mechanism of chromatin loop formation are not clear. While the majority of CTCF/cohesin-mediated loops are “hairpin loops” [3] with anchors containing CTCF motifs in convergent orientation, anchors with tandem CTCF motifs (i.e., CTCF motifs with the same orientation) can form “coiled loops” [3]. Moreover, multiple studies have shown that transcription factors (TFs) other than CTCF and cohesin may play an important role in loop formation [8, 9]. Recent experiments [7] support the loop extrusion model [10, 11], which suggests that structural maintenance of chromosome (SMC) proteins (e.g., cohesin or condensin) extrude chromatin until blocked by two CTCF proteins bound at convergent CTCF motif sites to form loops. Yet, it is not clear how and when CTCF proteins can prevent SMC proteins from extruding, and how SMC proteins can translocate along the chromatin at the rapid speeds observed in experiments [7, 12, 13]. In modeling insulator loops, a model has to learn DNA sequence patterns of CTCF-bound regions that can stop cohesin proteins from extruding because most CTCF-bound regions in fact do not form loops [14]. It becomes even more challenging to model insulator loops when DNA sequence of anchor regions contains multiple CTCF motifs with opposite orientations. Additionally, loops involve two loci, a start point and a stop point of the extrusion process, and loops can interconnect [3], so the model should be able to identify pairs of DNA fragments of regions that form loops. Recently, Hansen et al. [15] found evidence of RNA binding by CTCF to mediate a class of chromatin loops that show different DNA sequence patterns compared with RNA-independent loops. Thus, it is clear that there are more DNA sequence patterns besides CTCF motifs that are important for insulator loop formation and methods relying solely on the presence of CTCF motifs and/or CTCF motif orientation to predict or to model insulator loops are unlikely to do well.

Lollipop [16] is a computational method that attempts to predict CTCF-mediated loops from a range of genetic and epigenetic features. However, the method cannot be used to predict the impact of mutations on loops because it does not take into consideration specific DNA sequences and cannot account for sequence differences caused by mutations. CTCF-MP [17] also predicts CTCF-mediated loops from genetic and epigenetic features. It uses a model based on word2vec [18] to learn DNA sequence features and boosted trees to predict loops. CTCF-MP can account for sequence changes by mutations and can be used to predict the impact of mutations on loops. In spite of that, the word2vec model may not be able to learn complex DNA sequence features as evidenced by the inability of CTCF-MP to deal with loop anchors containing several CTCF motifs.

**Fig. 1:**
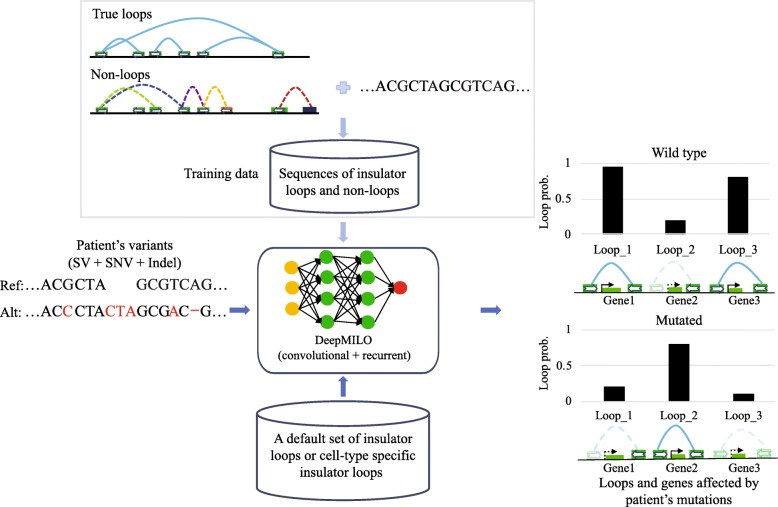
Overall approach for the identification of insulator loops and genes affected by mutations in patients. Anchors of insulator loops and non-loops are used to train DeepMILO to learn sequence features of insulator loops. The model is then applied to patients’ mutation data (i.e., structural variants, single nucleotide variants, and indels) to detect insulator loop changes associated with mutations. Insulator loops can be the set of default insulator loops from 4 cell lines GM12878, K562, MCF7, and Hela or a set of cell type-specific insulator loops provided by users

Convolutional neural network (CNN), a class of deep learning neural networks, has been successfully used to learn DNA sequence patterns such as those for DNA and RNA binding proteins [19], DNA methylation [20], or chromatin-profiling data [21]. Another class of deep neural networks is recurrent neural network (RNN), which is commonly used for learning tasks involving sequential data such as language translation and speech recognition. Yet, it has not been used widely on DNA sequence, which is a type of sequential data where the order and relationship between the bases are important for its function. While CNNs are good at capturing local patterns in sequences, RNNs like long short-term memory (LSTM) networks can capture long distance dependencies in sequential data. Here, we show that RNN can perform comparably with CNN model in learning DNA sequence patterns of anchors of insulator loops. Furthermore, they learned different features and combining their features delivered a better model compared to individual RNN or CNN models. Using features learned by a CNN and an RNN, we propose DeepMILO, a Deep learning approach for Modeling Insulator LOops, to learn DNA sequence features of insulator loops. The model can separate DNA sequences of insulator loop anchors bound by both CTCF and cohesin proteins from DNA sequences of CTCF ChIP-seq peaks bound by only CTCF and DNA sequences of regions without CTCF binding. DeepMILO can pair DNA sequences of anchors forming loops to distinguish insulator loops from different types of non-loops (i.e., fake loops) with high accuracy. Using DeepMILO, users can predict the impact of variants obtained by whole-genome sequencing of their samples on insulator loops from the cell type of interest (Fig. 1). We applied DeepMILO to 1834 patient samples from 12 International Cancer Genome Consortium (ICGC) cohorts to study how mutations are associated with insulator loop changes in different cancer types.

## Results

We first developed an “anchor model” to learn DNA sequence features of anchor regions of insulator loops. To pair two anchors to form an insulator loop, we built a model to distinguish left vs. right anchors of insulator loops using the learned features from the anchor model. This model is referred to as the “anchor orientation model.” The anchor and anchor orientation models were then combined to create DeepMILO with the capability of identifying anchors and pairing them with their partners to model DNA sequences of insulator loops. Given a pair of DNA sequences of anchors, this model outputs a number between 0 and 1 that can be interpreted as the loop probability or loop strength. The models were trained, validated, and tested with insulator loops from four cell lines GM12878, K562, MCF7, and Hela captured by cohesin (RAD21) ChIA-PET with PET peaks overlapping CTCF ChIP-seq peaks [5] or by CTCF ChIA-PET with PET peaks overlapping cohesin (RAD21) ChIP-seq peaks [3]. Anchors of insulator loops require co-occupancy of both CTCF and cohesin complex (SMC1, SMC3, RAD21, and either STAG2 or STAG1) [4, 22]; therefore, we only use loops with both CTCF and cohesin at their anchors. The data from chromosomes 7 and 8 was held out for testing, and the data from chromosome 16 was used for validation. Training datasets have approximately the same numbers of positive and negative samples, but test sets are imbalanced and include all possible negative samples. Due to the high numbers of true negatives, we used area under the precision-recall curve (AUPRC) approximated by average precision to measure the performance of the models.

### RNN and CNN features complement each other

**Fig. 2:**
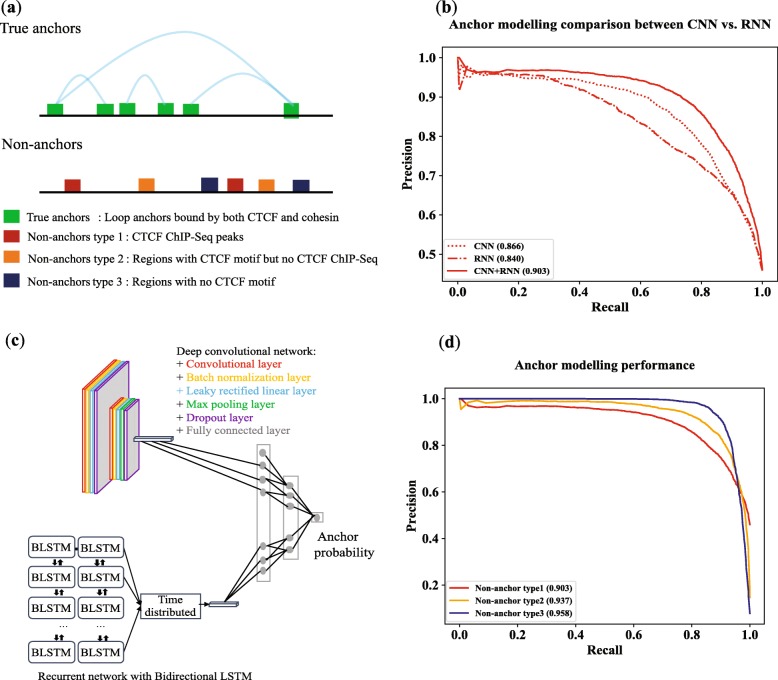
Datasets of anchors and non-anchors, and performance of models on these datasets. Numbers inside brackets in **c** and **d** are AUPRCs. **a** Different types of non-anchors used for training and testing. **b** The CNN model outperformed the RNN model, and their combination delivered the best performance for the non-anchor type 1 test set. Proportion of positive samples is 0.45. **c** Architecture of the anchor model consisting of a CNN and an RNN. **d** Performance of the anchor model (CNN + RNN) on test sets. Proportions of positive samples in datasets are 0.45, 0.12, and 0.075 for non-anchor types 1, 2, and 3, respectively

We built a CNN model and an RNN model to extract features directly from DNA sequences of anchors. The CNN model has three dilated CNNs with dilation rates of 1, 3, and 7 (see the “Methods” section). The RNN model uses bidirectional long short-term memory blocks [23] (see the “Methods” section). The models were trained separately to identify anchors of insulator loops. Individually, the CNN performed better than the RNN model (AUPRC of 0.866 for CNN vs. 0.840 for RNN) (Fig. 2b) for the task of separating sequences of insulator anchors (true anchors—Fig. 2a) from sequences of CTCF ChIP-seq peaks (non-anchor type 1—Fig. 2a). Both positive and negative samples have active CTCF motifs, but positive samples (true anchors) are also bound by cohesin proteins. High AUPRC values indicate that additional sequence rules govern the presence of insulator loop anchors besides the presence of actively bound CTCF motifs in a given cell type and our models were able to learn these rules.

We then built the anchor model by combining learned features from the RNN and CNN models (Fig. 2c). This model performed significantly better compared with the individual RNN and CNN models for the non-anchor type 1 test set (AUPRC of 0.903) (Fig. 2b). The result suggests that the RNN and CNN models learned different sets of features that complement each other for the task of identifying DNA sequences of insulator loop anchors.

### Performance of the anchor model on different test sets

The anchor model was built from learned features of a CNN and an RNN as discussed above (Fig. 2c). The model was tested with test sets containing anchors from chromosomes 7 and 8. We used three different test sets. They have the same set of true anchors as positive samples but differ in negative samples (Fig. 2a). The first test set contains negative samples from regions containing CTCF motifs overlapping CTCF ChIP-seq peaks but are not anchors of any insulator loop (non-anchor type 1). The second set consists of regions with CTCF motifs but not bound by CTCF protein as negative samples (non-anchor type 2). The third test set includes regions without CTCF motifs and peaks as negative samples (non-anchor type 3). The first test set is the most difficult one as many negative samples can have similar DNA sequence patterns with positive samples.

The results from the three test sets are shown in Fig. 2d. While the model performed well for the non-anchor type 1 test set achieving an AUPRC of 0.903 (also discussed above as part of Fig. 2b CNN + RNN), it performed even better for other test sets. For non-anchor type 2, i.e., when negative samples contain just CTCF motifs but are not bound by CTCF, the AUPRC is 0.937. And as expected, the model performed best for non-anchor type 3, i.e., when negative samples contain no CTCF motifs or peaks (AUPRC = 0.958). The results from non-anchor types 1 and 2 suggest that anchors of insulator loops have other sequence features besides the presence of CTCF motif and our model learned these features well.

### Motifs at insulator loop anchors

We then investigated what parts of sequences were being used by the CNN model for classification. Although it is expected that CTCF motif is enriched at insulator loop anchors, this analysis was done to reveal if other TF motifs are also enriched. We used DNA sequences of 700 true anchors and performed class activation map (CAM) visualization [24] to generate heatmaps showing how different parts of the sequences were being used by the CNN model. CAM assigned a score to each base of a sequence indicating how the base activated the class of the sequence (as an anchor). For each anchor, we performed peak calling (details in the “Methods” section) to identify regions of high intensity in the CAM signal output (referred to as CAM peaks). On average, there are 29 CAM peaks per anchor. We aggregated positions of CAM peaks relative to anchor centers over the 700 anchors (Additional file 1: Fig. S1) and found that CAM peaks are distributed relatively evenly across the 4000 bases of anchors. This result shows that the CNN model used many parts of sequences to distinguish anchors from non-anchors. We then used Analysis of Motif Enrichment (MEME Suite) [25] to identify known motifs (HOCOMOCO v11 FULL) enriched at CAM peaks (*E* value < 0.0001) and found 38 motifs (the list of motifs is shown in Additional file 2: Table S1). As a key element of insulator anchors, CTCF motif is significantly enriched. In addition, we note that motifs of some other members of the zinc finger TF family (ZN770, ZN121, ZN335, and IKZF1) appear significantly enriched. It is interesting that ZFX is among the enriched motifs. This result is in concordance with the previous analysis in [16].

### Anchor orientation model to distinguish left and right anchors

While most insulator loops contain CTCF motifs in convergent orientation at the paired anchor regions, an anchor element can contain several CTCF motifs with different orientations. Moreover, the paired anchors of some loops contain tandem CTCF motifs [3]. Therefore, CTCF motif orientation alone cannot be used to distinguish the two anchors of insulator loops. We used the learned features of the anchor model to build the anchor orientation model to distinguish left anchors from right anchors. This model shares its features with the CNN and RNN of the anchor model. DNA sequences of anchors in chromosomes 7 and 8 were used to test the model regardless of the number and orientation of their CTCF motifs. The results show that the left and right anchors can be well separated with an AUPRC of 0.96. We note that 49.4% of anchors in the test set contain multiple CTCF motifs, which cannot be handled by methods relying on CTCF motif orientation. While models like CTCF-MP [17] or Lollipop [16] require CTCF motif orientation in their input, our results show that the anchor orientation model has learned features to distinguish left and right anchors of loops de novo from the sequence.

### Loop model for learning features of pairs of DNA sequences of anchors of insulator loops

**Fig. 3:**
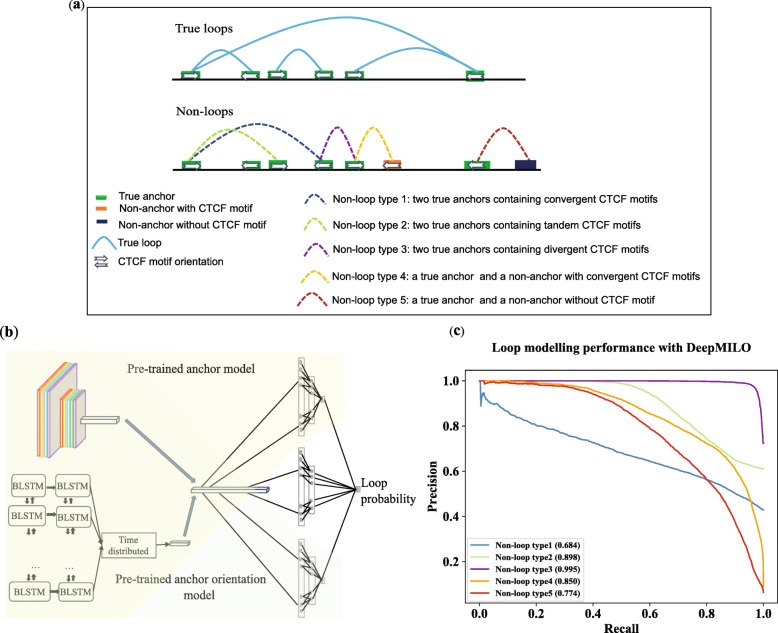
Datasets of loops and non-loops, and performance of DeepMILO on these datasets. Numbers inside brackets in **c** are AUPRCs. **a** Different types of non-loops for training and testing the loop model. **b** Details of DeepMILO; combining pretrained anchor model and pretrained anchor orientation model helped training to converge faster. **c** Performance of DeepMILO on different test sets. Proportions of positive samples are 0.43, 0.61, 0.72, 0.072, and 0.063 for non-loop types 1, 2, 3, 4, and 5 datasets, respectively

As loops often interconnect and one anchor can be involved in several loops [3], pairing anchors forming loops is not trivial. We combined the anchor model and anchor orientation model to build DeepMILO to model insulator loops and effects of mutations on these loops (Fig. 3b).

To ensure a fair evaluation of the model performance, five different types of test sets were created to test the model. They have the same set of positive samples from true insulator loops. However, their negative samples are different as illustrated in Fig. 3a. We also constrained the distance between two anchors of negative samples (non-loops) by the 75th percentile of distances between paired anchors of true insulator loops. The negative samples in the first type of test set consist of non-loops formed from two true anchors with convergent CTCF motifs (non-loop type 1). This type of test set is the most difficult one as many non-loops possess similar patterns with true loops. We note that some of the non-loops in this set could be actual loops that were not captured in ChIA-PET experiments. The second type of test set includes non-loops from two true anchors but containing tandem CTCF motifs (non-loop type 2). The third type of test set contains non-loops from two true anchors with divergent CTCF motifs (non-loop type 3). The fourth type of test set consists of non-loops with one true anchor and one non-anchor containing a CTCF motif in convergent orientation with the CTCF motif at the true anchor (non-loop type 4). The fifth type of test set includes non-loops with one true anchor and one non-anchor containing no CTCF motif or peak (non-loop type 5). The results from test sets are shown in Fig. 3c.

For the most difficult test set, the non-loop type 1 test set, DeepMILO achieved an AUPRC of 0.684. A naïve model using CTCF motif orientation only would achieve an AUPRC of ~ 0.429 (proportion of positive samples). For the non-loop type 2 and 3 test sets, DeepMILO performed very well (AUPRCs of 0.898 and 0.995, respectively). For the non-loop type 4 test set, DeepMILO also performed well with an AUPRC of 0.850. This test set contains non-loops with paired anchors containing convergent CTCF motifs so that they are relatively difficult to classify. Moreover, the proportion of positive samples is very small (0.073). Models relying solely on CTCF motif orientation would achieve an AUPRC of ~ 0.073 for this test set. Lastly, DeepMILO performed very well on non-loop type 5 test set with one non-anchor without CTCF motif (AUPRC of 0.774). We compared DeepMILO with a model based on word2vec [18] and boosted trees similar to CTCF-MP [17] using DNA sequence features (Additional file 3).

Next, we checked how well tandem loops (insulator loops with the same orientation of CTCF motifs at anchors) are satisfied. To determine if an insulator loop is satisfied in the test set, we picked the loop probability threshold that yielded equal precision and recall in the validation set. There are 949 tandem insulator loops in the test set, and 65% of them are satisfied (i.e., predicted as positive) based on prediction from our model. This result demonstrates that our model has learned patterns of tandem insulator loops that cannot be learned by models relying on convergent CTCF motif orientations.

We provide DeepMILO as a software tool to predict the impact of sequence variants on insulator loops. Given a set of variants, DeepMILO evaluates their impact on a set of ~ 74,000 insulator loops from four cell lines GM12878, K562, Hela, and MCF7 (default option) or a set of insulator loops from a cell type of interest (input by the user) and outputs loop probabilities with and without variants (Fig. 1). Users can then compare loop probabilities to identify altered loops. It is also possible to evaluate the effects of individual variants on insulator loops to identify functional variants.

### Validation of DeepMILO with known loop-disrupting deletions

We next tested DeepMILO on two known deletions that disrupt insulator loops. Hnisz et al. [5] introduced two deletions found in T-ALL patients using CRISPR/Cas9 at anchors of insulator loops containing the oncogenes *TAL1* and *LMO2*. The authors showed that the deletions increased interactions between enhancers and promoters that were insulated by anchor elements in the wild type cells leading to upregulation of *TAL1* and *LMO2* in the edited cells. We evaluated the impact of these two deletions on insulator loops. Among the ~ 74,000 insulator loops, two loops cover the *TAL1* gene and 19 loops cover the *LMO2* gene (Additional file 1: Fig. S2). The deletion in neighborhood of *LMO2* is of length 25 kb (Additional file 1: Fig. S2). DeepMILO predicted loop probabilities of the 19 loops after the deletions are reduced to 0.0008 from 0.24–0.91. The result indicates that the model correctly predicted the impact of the deletion on insulator loop disruption. For the case of *TAL1*, the deletion is 400 bp (Additional file 1: Fig. S2 c). With the deletion, DeepMILO predicted the loop probabilities decreased to less than 0.26 from 0.89 and 0.91, suggesting that the insulator loops are weakened or disrupted. These results are consistent with the experimental results in [5].

Given the large size of the two deletions, it is expected that they can disrupt the associated insulator loops. To test the sensitivity of DeepMILO with small mutations (single nucleotide variants and indels) and if small mutations are sufficient to disrupt insulator loops, we simulated 400 small deletions of one base for every position of the deletion related to *TAL1* and used DeepMILO to predict the impact of these small deletions. Comparing loop probabilities without and with individual mutations, we identified 11 consecutive mutations at the center of the deletion with highest reductions in loop probability (Additional file 1: Fig. S2 c). We find that the DNA sequence of these 11 positions matches the CTCF motif well (*p* value = 1.96e−03, Additional file 1: Fig. S2 c). The reduction in loop probability is as high as 0.70, indicating that small mutations of one base could result in significant impact on insulator loops and that DeepMILO is sensitive to small mutations. These results demonstrate that DeepMILO can be used to identify the exact bases among large deleted sequence whose alteration would be associated with loop disruption. We note that these results were based on individual single nucleotide mutations and therefore do not rule out the possibility of small mutations of several bases or combinations of small mutations outside CTCF motif causing significant effects on insulator loops.

### Identification of disrupted insulator loops in 1834 patient samples from twelve cancer cohorts

**Fig. 4:**
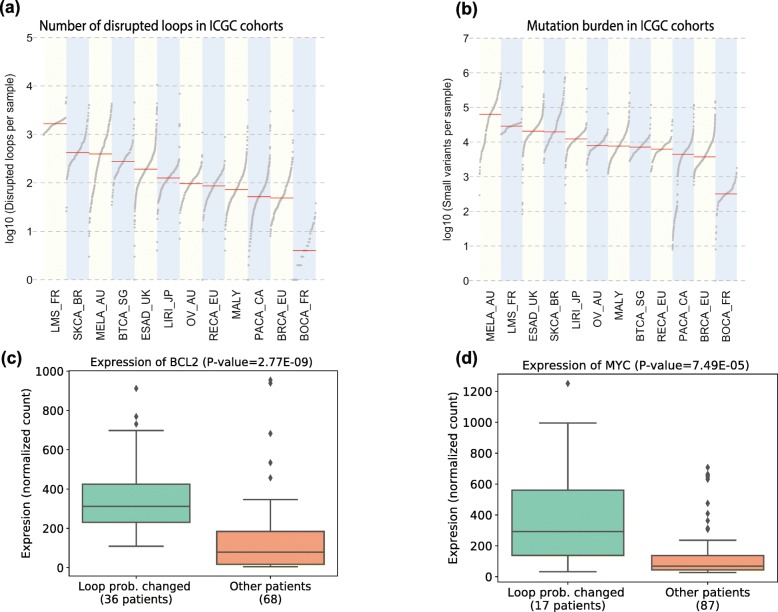
Application of DeepMILO on patient samples. LMS, soft tissue cancer—leiomyosarcoma; SKCA, skin adenocarcinoma; MELA, skin cancer; BTCA, biliary tract cancer; ESAD, esophageal adenocarcinoma; LIRI, liver cancer; OV, ovarian cancer; RECA, renal cell cancer; MALY, malignant lymphoma; PACA, pancreatic cancer; BRCA, breast ER+ and HER2− cancer; BOCA, soft tissue cancer—Ewing sarcoma. **a** Number of disrupted insulator loops in ICGC cohorts. **b** Mutation burden in ICGC cohorts. **c**, **d** Oncogenes *BCL2* and *MYC* show differential expression between patients with decrease in loop probability and other patients (MALY cohort, Wilcoxon signed-rank test)

The majority of somatic variants in cancer cells reside in non-coding regions [26]. Some of these variants can affect 3D chromatin structures, which can in turn activate proto-oncogenes [5, 27]. We sought to apply DeepMILO to somatic variants from whole-genome sequences of twelve ICGC cohorts of cancer patients with a total of 1834 samples to identify disrupted insulator loops associated with non-coding variants. We only consider small variants (single nucleotide variants and indels) because their effect is difficult to predict and not all cohorts have structural variant data. To determine if a loop probability reduction is significant, we use the 90th percentile of all loop probability reductions across all samples and insulator loops as a cutoff threshold (distribution of probability reductions shown in Additional file 1: Fig. S3). Among ~ 74,000 insulator loops, there are 672 loops disrupted in at least 10% of patients (183 patients). Mutational burden is strongly correlated with the numbers of disrupted loops (Fig. 4 a, b; 0.87 Spearman’s rank correlation between medians). Thus, in general, more mutations are associated with more disrupted insulator loops. This result indicates that cancers with higher mutational burden are likely to have higher chromatin instability. Leiomyosarcomas (LMS_FR cohort), a type of soft tissue sarcoma, is an exception. It has the most disrupted insulator loops though it has far fewer number of mutations compared to melanoma (MELA_AU cohort) (median number of mutations and disrupted loops are 28,868 and 1660 in LMS_FR, respectively, and 63,571 and 420 in MELA_AU, respectively). This may be related to the transcriptional changes associated with tumor evolution in leiomyosarcomas [28].

Next, we looked for genes with expression changes associated with disrupted insulator loops when matching RNA-seq data is available for a large number of samples and there are disrupted insulator loops in at least 10% of patients (to ensure sufficient sample sizes for statistical testing). In the ICGC cohorts, only malignant lymphoma (MALY) cohort satisfies the conditions. The cohort has 241 patients with whole-genome sequences, and 104 patients also have matching RNA-seq data. We identified 18 insulator loops disrupted in at least 10% of patients (24/241 patients). There are six genes inside these 18 loops. Among these six genes, there are four three cancer genes: *MYC*, *BCL2*, and *KDSR*. *BCL2* and *MYC* show differential expression in patients with decrease in loop probabilities vs. the other patients (Fig. 4 c, d; Wilcoxon signed-rank test). We did not find structural variants or significant differences in copy number for *MYC* and *BCL2*, suggesting that changes in the strength of insulator loops could contribute to their upregulation.

## Discussion and conclusions

We present DeepMILO for modeling insulator loops and for predicting the effects of variants on these loops. DeepMILO has learned sequence features of insulator loops beyond the presence and orientation of CTCF motifs. It can identify insulator loops with high AUPRC. DeepMILO uses features learned by a CNN model and an RNN model. We show that RNN can perform comparably with CNN in learning DNA sequence patterns of insulator loop anchors and that combining learned features of RNN and CNN models delivers a better model compared with individual models. Furthermore, we find that small mutations of one base can result in significant impact on 3D insulator loops and that our model is sensitive enough to predict the impact of such small mutations. Using DeepMILO, we identified insulator loops predicted to change in multiple cancer patients and genes affected by these loops. Our model suggests a possible new mechanism for upregulation of *BCL2* and *MYC* in malignant lymphoma via alteration of insulator loops. DeepMILO can be extended for other 3D structures such as enhancer-promoter loops and TADs to identify the non-coding variants altering 3D chromatin structures in general. Identification of these variants together with altered 3D structures can provide insights into the mechanism of aberrant gene expression in disease.

## Methods

### Data preparation

We used CTCF/cohesin insulator loop data of the cell lines GM12878, K562, Hela, and MCF7 to train and test our models. These loops were captured by cohesin (RAD21) ChIA-PET with PET peaks overlapped by CTCF ChIP-seq peaks [5] or captured by CTCF ChIA-PET with PET peaks overlapped by cohesin (RAD21) ChIP-seq peaks [3]. CTCF and RAD21 ChIP-seq data were obtained from ENCODE [29].

Anchors of loops were normalized as follows. Two anchors *b*_1_ and *b*_2_ with lengths *l*_1_ and *l*_2_ are considered as equal and merged into one anchor if their overlap is larger than 0.9 × *l*_1_ or 0.9 × *l*_2_. After merging equal anchors, we obtained a set of normalized anchors and loops were formed from these anchors. Anchors were then trimmed off or expanded to have a length of 4000 bases (~ median length of anchors captured by cohesin ChIA-PET) centered at their peaks. A sequence is converted into a one-hot encoding matrix of size [4000 × 5]. Due to the computationally intensive nature of LSTM, only 800 bases centered around the middle points of sequences were inputted into the RNN model.

Data from chromosomes 7 and 8 was held out for testing, and data from chromosome 16 was used for validation. We checked performance of our models with a test set from chromosomes 1 and 14, and found that performance of models was similar (data not shown), indicating that data from chromosomes 7 and 8 was not easier for models. Negative anchor samples containing CTCF ChIP-seq peaks were derived from CTCF ChIP-seq signal and were centered around the middle points of peaks. Negative anchor samples containing CTCF motifs were calculated from the location of CTCF motifs detected by FIMO [25] using a *p* value threshold of 0.00005, and they were also centered around CTCF motifs. Negative anchor samples without CTCF motifs are all regions of 4000 bases that do not overlap any CTCF motif.

Positive and negative samples were balanced in training and validation. Test sets are imbalanced and contain all possible negative samples. For each sequence, its complementary reverse sequence was also included to increase the amount of training data. Additionally, by training the model with complementary reverse sequences, the strand of sequences can be ignored when running the model. In preparation of the data to train the “anchor orientation model,” we removed anchors that can be considered as both left and right anchors. Left and right anchors were considered as negative and positive samples, respectively.

### CNN, RNN, and anchor model for learning features of anchors

We developed a deep convolutional neural network (CNN) and a recurrent neural network (RNN) with bidirectional long short-term memory cells to learn DNA sequence patterns of anchors of insulator loops. Learned features from these two models were combined to build the anchor model to distinguish anchors from non-anchors.

DNA sequences of anchor and non-anchor regions were used to train the models. In training, validation, and test sets, positive samples include true anchors and negative samples consist of the three types of non-anchors with a ratio of 50%:30%:20% for non-anchor types 1, 2, and 3, respectively.

A DNA sequence was converted to a one-hot encoding matrix with *m* rows and 5 columns, where *m* is the length of the sequence and 5 columns corresponding to 5 bases A, C, G, T, and N. The models were trained to output a number in the range of [0, 1] that can be interpreted as anchor probability of a given DNA sequence. It is expected that probabilities of DNA sequences of anchor regions are closer to 1 and probabilities of DNA sequences of non-anchor regions are close to 0.

The CNN and RNN models were trained separately, and their features were later combined for the anchor model. The same training and validation datasets were used to train the three models. The CNN model has two convolutional layers. The first layer of the network is a convolutional layer with 256 filters of size [17 × 5]. Filters scan through input sequences and are applied to each allele separately. This layer is followed by a batch normalization [30], a leaky rectified linear, and a dropout layer [31]. The batch normalization layer stabilizes the output from the first layer before it goes through the leaky rectified linear layer and speeds up the optimization during training. The dropout layer prevents the network from overfitting. Our experiments found that a negative slope coefficient of 0.2 for the leaky rectified linear function and a dropout rate of 0.3 produced the best result. Following these layers are three parallel dilated convolutional layers with dilation rates of 1, 3, and 7. They have 512 filters of size [5 × 1], and their outputs are concatenated. These dilated convolutional layers are supposed to combine features from the first convolutional layers to learn higher level features. Our experiments showed that these convolutional layers allowed us to achieve better performance with less training time. However, adding more convolutional layers did not yield a clearly better performance while significantly increasing the complexity of the model. The dilated convolutional layers are followed by a batch normalization, a leaky rectified linear, a global max pooling, and a dropout layer. The global max pooling layer is intended to capture if the input contains specific patterns learned by filters of convolutional layers. The output from these layers is then concatenated and inputted into two fully connected layers with 256 and 128 nodes, respectively. The last layer of the model is a sigmoid activation node that outputs a probability of the input sequence as anchor of an insulator loop. We used a binary cross entropy loss function as objective function. And it was minimized using the RMSprop algorithm.

The RNN model consists of two stacked bidirectional LSTM layers (BLSTM) that can capture long-term dependency in long sequences. Bidirectional LSTM processes sequences in both directions, forward and backward directions, and therefore often captures the context better. Each BLSTM layer has 64 hidden units. A dropout layer with a dropout rate of 0.2 follows each layer to prevent overfitting. Output from BLSTM layers is inputted into a time distributed layer. Following the time distributed layer are fully connected layers with the same settings as in the CNN model.

To combine the learned features of the CNN and RNN models, their fully connected layers and output layers were stripped down after training and outputs from the remaining layers of the two models were concatenated and inputted into two new fully connected layers with 512 and 256 nodes as in the CNN and RNN model. The models were implemented in Keras (https://keras.io).

### Peak calling to identify CAM peaks

Based on the nucleotide score signal of the class activation map (CAM) [24], we performed peak calling to identify the bases with high intensity. We fitted a smooth curve by non-parametric local polynomial regression (LOESS) using *α* = 0.4 grade of smoothness. This curve captured the trend of focal high intensity because the fitting was weighted toward the nearest surrounding score values. Then, we called peaks for 40 bp sliding windows.

### Anchor orientation model to distinguish left and right anchors

We constructed the anchor orientation model from learned features of the anchor model. The two fully connected layers of the anchor model were replaced by two new fully connected layers of 256 and 128 nodes, respectively. We then trained this model to distinguish left and right anchors of insulator loops. Left and right anchors were considered as negative and positive samples, respectively. The model is expected to produce values close to 0 for left anchors and values close to 1 for right anchors.

### Loop model for learning sequence patterns of insulator loops

We built DeepMILO by combining learned features of the anchor and anchor orientation models as shown in (Fig. 3b). The output layer of the anchor model was removed, and the two fully connected layers were replaced by two new layers with the same settings to make a new sub-model. Then, the output from this new sub-model was concatenated with outputs from the anchor and anchor orientation model. The loop model uses sequence features of anchors and predicted outcomes from the anchor model and the anchor orientation model for its prediction. In training, validation, and test sets, positive samples are true insulator loop and negative samples consists of non-loop types 1, 2, 3, 4, and 5 with a ratio of 50%:10%:10%:20%:10%, respectively.

## Supplementary information

**Additional file 1: Figure S1.** Relative positions of CAM peaks across anchors. **Figure S2.** Validation of the loop model with known deletions that disrupt insulator loops. **Figure S3.** Reductions in loop probability because of mutations predicted by DeepMILO.

**Additional file 2: Table S1.** Motifs found in loop anchors.

**Additional file 3.** Comparison with CTCF-MP.

**Additional file 4.** Review history.

## Data Availability

Source code is available in Github and Zenodo under MIT license [32, 33]. Cohesin (RAD21) ChIA-PET data is available from [5]. CTCF ChIA-PET data is available from [3]. CTCF and RAD21 ChIP-seq data were obtained from ENCODE [29].
