# Supplementary material for: DeepMILO: a deep learning approach to predict the impact of non-coding sequence variants on 3D chromatin structure
Source: Genome Biol. 2020 Mar 26;21:79. doi: 10.1186/s13059-020-01987-4 (PMC7098089; doi:10.1186/s13059-020-01987-4)
Supplement: Supplementary file 1 — Additional file 1: Figure S1. Relative positions of CAM peaks across anchors. Figure S2. Validation of the loop model with known deletions that disrupt insulator loops. Figure S3. Reductions in loop probability because of mutations predicted by DeepMILO. [file 13059_2020_1987_MOESM1_ESM.docx]

**Supplementary figures**

**Fig. S1 Relative positions of CAM peaks across anchors.**

CAM peaks, where the CNN model focused on to identify anchors, distribute relatively evenly across the 4,000 bases of anchors.

**Fig. S2 Validation of the loop model with known deletions that disrupt insulator loops.**

(**a**) Insulator loops cover LMO2 gene. The deletion decreased loop probabilities from [0.24-0.91] to 0.0008 (**b**) Insulator loops cover TAL1 gene. Loop probabilities decreased from 0.89 and 0.91 to 0.25 and 0.26 because of the deletion. (**c**) Impact of 400 small deletions on a loop containing TAL1.

**Fig. S3 Reductions in loop probability because of mutations predicted by DeepMILO**

Majority of small mutations have little impact on insulator loops however many mutations caused dramatic changes in loop probability.
