# Supplementary material for: DeepMILO: a deep learning approach to predict the impact of non-coding sequence variants on 3D chromatin structure
Source: Genome Biol. 2020 Mar 26;21:79. doi: 10.1186/s13059-020-01987-4 (PMC7098089; doi:10.1186/s13059-020-01987-4)
Supplement: Supplementary file 2 — Additional file 2: Table S1. Motifs found in loop anchors. [file 13059_2020_1987_MOESM2_ESM.docx]

**Motifs found in loop anchors**

**Table S1: Motifs found by Analysis of Motif Enrichment (AME) from MEME suite in regions used by the CNN model to identify loop anchors**

| rank | motif_ID | p-value | adj_p-value | E-value |
| --- | --- | --- | --- | --- |
| 1 | ZN770 | 3.32E-112 | 1.79E-109 | 1.38E-106 |
| 2 | PITX2 | 4.07E-79 | 1.40E-75 | 1.08E-72 |
| 3 | ZN121 | 1.02E-63 | 2.27E-61 | 1.74E-58 |
| 4 | IKZF1 | 2.91E-50 | 2.45E-46 | 1.88E-43 |
| 5 | MEF2D | 3.39E-40 | 4.56E-37 | 3.51E-34 |
| 6 | FUBP1 | 8.35E-39 | 3.23E-35 | 2.48E-32 |
| 7 | HMGA1 | 4.97E-37 | 7.65E-33 | 5.88E-30 |
| 8 | MEF2C | 8.36E-35 | 1.58E-31 | 1.22E-28 |
| 9 | MEF2A | 1.19E-34 | 2.40E-31 | 1.84E-28 |
| 10 | E2F3 | 4.23E-34 | 1.83E-30 | 1.41E-27 |
| 11 | ETV7 | 3.73E-32 | 3.28E-28 | 2.52E-25 |
| 12 | E2F7 | 1.08E-31 | 3.51E-28 | 2.70E-25 |
| 13 | PRDM6 | 8.13E-32 | 4.59E-28 | 3.53E-25 |
| 14 | TAF1 | 5.39E-31 | 8.88E-28 | 6.83E-25 |
| 15 | PAX5 | 6.49E-30 | 1.55E-27 | 1.19E-24 |
| 16 | TBX21 | 2.94E-31 | 1.64E-27 | 1.26E-24 |
| 17 | FOXP1 | 3.38E-31 | 2.66E-27 | 2.04E-24 |
| 18 | RARB | 4.87E-29 | 6.75E-26 | 5.19E-23 |
| 19 | OTX1 | 2.65E-29 | 1.02E-25 | 7.87E-23 |
| 20 | FOSB | 6.59E-29 | 1.96E-25 | 1.51E-22 |
| 21 | E2F6 | 3.07E-28 | 9.01E-25 | 6.93E-22 |
| 22 | ETV6 | 3.69E-28 | 2.74E-24 | 2.11E-21 |
| 23 | CTCF | 1.49E-26 | 3.79E-24 | 2.92E-21 |
| 24 | MLX | 1.60E-26 | 3.85E-24 | 2.96E-21 |
| 25 | KLF14 | 2.24E-26 | 1.38E-23 | 1.06E-20 |
| 26 | FOXG1 | 1.17E-26 | 2.30E-23 | 1.77E-20 |
| 27 | ZFX | 9.27E-27 | 2.49E-23 | 1.92E-20 |
| 28 | MEF2B | 4.49E-26 | 8.23E-23 | 6.33E-20 |
| 29 | BACH1 | 3.21E-25 | 3.26E-22 | 2.51E-19 |
| 30 | ZN335 | 9.45E-26 | 5.68E-22 | 4.37E-19 |
| 31 | MYCN | 1.92E-24 | 1.50E-21 | 1.15E-18 |
| 32 | ITF2 | 3.76E-25 | 1.75E-21 | 1.34E-18 |
| 33 | RARA | 1.93E-24 | 7.26E-21 | 5.58E-18 |
| 34 | SOX10 | 3.42E-24 | 1.89E-20 | 1.45E-17 |
| 35 | MAX | 1.07E-23 | 2.25E-20 | 1.73E-17 |
| 36 | P63 | 1.93E-23 | 5.72E-20 | 4.40E-17 |
| 37 | WT1 | 3.42E-23 | 1.47E-19 | 1.13E-16 |
| 38 | XBP1 | 1.10E-22 | 1.75E-19 | 1.34E-16 |
