# Supplementary material for: DeepMILO: a deep learning approach to predict the impact of non-coding sequence variants on 3D chromatin structure
Source: Genome Biol. 2020 Mar 26;21:79. doi: 10.1186/s13059-020-01987-4 (PMC7098089; doi:10.1186/s13059-020-01987-4)
Supplement: Supplementary file 3 — Additional file 3. Comparison with CTCF-MP. [file 13059_2020_1987_MOESM3_ESM.docx]

**Comparison with CTCF-MP**

**Implementation of a classifier using word2Vec and boosted trees**

Following the CTCF-MP model in [1] we implemented a classifier using word2Vec and boosted trees to identify loops using DNA sequence only. CTCF-MP utilizes both genetic and epigenetic features (including CTCF ChIP-seq) to make prediction, however, using only DNA sequence features from word2vec, it predicted CTCF ChIA-PET loops with AUROC of 0.796 [1]. We implemented a similar model that takes DNA sequence features only as input to compare with DeepMILO. Boundaries were represented by DNA sequences of size 520 bases centered around their middle points. Our implementation achieved a comparable average AUROC of 0.794 in a 10-fold cross-validation using the same dataset by CTCF-MP [1]. We then trained the classifier with the same training datasets used for DeepMILO. And the classifier was tested with the five test sets to compare with DeepMILO. To improve performance of this model, we also tried to increase the anchor size to allow the model to capture more context of the data but found that the model generally performed worse with longer anchor sizes.

**Comparison between DeepMILO and CTCF-MP**

DeepMILO performed significantly better than CTCF-MP (see the table below for side-by-side comparison). However, it is worth to note that the design goals of CTCF-MP and DeepMILO are different and test sets used in our study have different characteristics from the test sets used in CTCF-MP’s study [1]. CTCF-MP was designed to predict CTCF loops using both epigenetic features (e.g. CTCF ChIP-seq) and sequence-based features. It requires location and orientation of CTCF motif in the input and assumes that there is only one CTCF motif in anchor regions. On the other hand, DeepMILO focuses on automatically learning sequence features of insulator loops, which requires both CTCF and cohesin at their anchors, and on predicting the effect of mutations on insulator loops. Therefore, DeepMILO’s input consists of DNA sequences only. Furthermore, training and test data in our study contains anchors with multiple CTCF motifs and was constructed to train models to identify insulator loops with both CTCF and cohesin at their anchors. These differences in design goals and the difficulty of the test data led to the poor performance of CTCF-MP. Our comparison aims at demonstrating that models such as CTCF-MP are incapable of learning complex sequence features to effectively predict the effect of mutations on insulator loops.

**Performance comparison between DeepMILO and Word2Vec + boosted trees.**

DeepMILO outperformed the model based on word2vec + boosted trees. Average precision is used to measure performance of the two methods.

|  | DeepMILO | Word2Vec + boosted trees  (CTCF-MP) |
| --- | --- | --- |
| Non-loop type 1 | 0.684 | 0.502 |
| Non-loop type 2 | 0.898 | 0.611 |
| Non-loop type 3 | 0.995 | 0.722 |
| Non-loop type 4 | 0.850 | 0.109 |
| Non-loop type 5 | 0.774 | 0.062 |

[1] R. Zhang, Y. Wang, Y. Yang, Y. Zhang, and J. Ma, “Predicting CTCF-mediated chromatin loops using CTCF-MP,” *Bioinformatics*, vol. 34, no. 13, pp. i133–i141, Jul. 2018.
