# Supplementary material for: DeepMILO: a deep learning approach to predict the impact of non-coding sequence variants on 3D chromatin structure
Source: Genome Biol. 2020 Mar 26;21:79. doi: 10.1186/s13059-020-01987-4 (PMC7098089; doi:10.1186/s13059-020-01987-4)
Supplement: Supplementary file 4 — Additional file 4. Review history. [file 13059_2020_1987_MOESM4_ESM.docx]

Review History

**First round of review**

**Reviewer 1**

**Comments to author:**

Summary
The authors present a novel computational method for estimating the probability of chromatin looping based on DNA sequence, and also for pinpointing mutations (not specific to CTCF motifs) that can alter insulator loops. They point out that their method is similar in its functionality to Lollipop and -mainly- CTCF-MP.
They use 2 types of neural networks, a 2-layer ConvNet and a LSTM-based RNN. First, they train them to predict boundary/anchors (boundary model) and from the surrounding sequence and then boundary orientation (orientation model) from the features they learned during the first part. Finally, their method (DeepMILO) consists of training and ensemble network based on the features learned during the first stage and the predictions of the other two networks to predict the probability that a given pair of sequences form a loop (loop score).
To train their model, they downloaded from ENCODE CTCF/cohesin chromatin loops for the GM12878 and K562 cell lines. These loops were captured by cohesin (RAD21) ChIA-PET and their PET peaks were overlapped with CTCF ChIP-seq peaks. The input to the model is pairs of sequences (4000 for ConvNet, 800 for RNN) and the output loops scores for a predefined set of loops. They split the data into train, validation, and test data-sets.
-- The validation data-set consisted of loops from chromosome 16 of the GM12878 cell line.
-- The testing data-set consisted of chromosomes 7 and 8 from both cell-lines
-- The training data-set consisted of the rest of the chromosomes for the GM12878 cells.
The test set was further divided into categories of different levels of difficulty of detection.
The authors validated their findings with two experiments.
First, the calculated the loop score changes induced by known loop-disrupting deletions (near TAL1 and LMO2) and noticed significant drops in estimated looping probabilities. They further noticed that main contributors were the 11 positions that matched the CTCFL motif.
Second, they predicted changes in loop probabilities induced by non-coding variants in a set of highly mutated patient samples from ICGC. They noticed that mutational burden is positively correlated (spearman 0.27) with changes in the loop score. Then they found loops that are significantly decreased in 10/241 of the patients and they checked for differential expression between the genes in these loops but they only found 1 out of 14 genes to be DE.
Overall this study start with an interesting idea, but its application is very limited and the models are not tested in a variety of cell lines (possibly due to lack of data). Also, the utility of the model is questionable since when tested in a large patient cohort with high mutational burden, it only produced minimal insights. Perhaps a pan-cancer analysis might have shown more promising results.

Major Comments
-- The authors claim that:
"We developed a powerful method, DeepMILO, for modelling insulator loops and for predicting the effect of variants on these loops. DeepMILO has learned sequence features of insulator loops that are beyond the presence and orientation of CTCF motifs"
This statement seems to imply that DeepMILO can predict non-CTCF related loops despite the fact that it has never seen any non-CTCF related positive example (since the training examples were RAD21 ChIA-PET loops intersected with CTCF ChIP-seq). This reviewer feels that the authors should clarify what is the scope and limitation of their model more adequately. If they believe that their model can consistently identify non-CTCF loops, they should perform some feature extraction analysis, similar to the one they did for their 1 validation experiment, in order to identify which non-CTCF related features are predictive according to their model.
-- CTCF-MP reports much higher AUPRs for the same cell lines. Can the authors explain why they get so different results? It would also be interesting if the authors could extend their analysis to include all 4 cell-lines utilized by CTCF-MP and also test how well their method generalizes across all cell lines.
-- The second validation experiment (patient data) is not very convincing. Only one (BCL2) of the 14 gene seems to be differentially expressed. The reported p-value is high enough to suggest that it could have arisen by chance in 14 tries. More importantly, BCL2 is known to be deregulated by a chromosomal translocation in lymphomas which could potentially induce multiple transcription-regulating events such as the landing position, but the authors only check for CNVs between the groups. More direct evidence, if possible an analysis on paired sequence and ChIP-seq data, is required to establish the clinical relevancy of the method.
-- When testing on the K562 cell line, it is important to also test on chromosomes other than chr7 and chr8 to exclude the possibility that these two chromosomes have more easily identifiable loops. Also detailed metrics in each chromosome should be reported to assess the chromosome-to-chromosome variability in performance
-- More importantly, more cell lines should be tested. If no other RAD21 ChIA-PET exists, the authors can be creative and think of other options, e.g. test Hi-C based TAD/sub-TAD boundary loops.
Minor Comments
-- The color of the nodes in then FC-layer of the neural nets is a bit misleading, it appears as if they are different types of nodes (figures 2b, 3b)
-- The authors may want to use the term loop "anchor" instead of "boundary", as "boundary" can easily be confused with TAD boundaries.

**Reviewer 2**

**Comments to author:**

Trieu and Khurana presented a new method to predict the effects of variants on chromatin loops, and therefore possible target genes. The idea of using 3D genome to help decipher variants has been interested in medical research for long time, and a lot of cases have been reported in the literature. However, a tool to systematically address this question remains rare. In that sense, the present work was trying to target an important and interesting question in the field. There are several issues need to be clarified.

Major
1.     The author compared DeepMILO with CTCF-MP. However, the assessment could be misleading.
a)     CTCF-MP was designed and trained by CTCF ChIA-PET data along. In CTCF-MP, a preferred convergent orientation of CTCF is a strong signal for loop prediction. However, in this paper, the authors were using ChIA-PET data of RAD21 to train and test the tools. Moreover, CTCF-MP asked for 520bp should be the flanking of motif center, not the middle of boundaries which were used in this paper. It may unfair to CTCF-MP, although the peak of CTCF and RAD21 are largely overlapped. And overlapping of two datasets could bring more information, per se.
b)     More important, there are no CTCF motif, or binding, or incorrect orientation in type 2,3,4 and 5. Thus, it is unfair to CTCF-MP in those testing data sets.
So, why don't use ChIA-PET of CTCF directly?
2.     The false positive could be over-estimated, as ChIA-PET only captures loops mediated by the targeted proteins, while the predicted loop may not necessarily be mediated by that protein.
3.     The simulations on Hnisz et al data simply means that CTCF motif plays a major role in the loop prediction. Or, in another word, the loops predicted by DeepMILO should largely be CTCF loop, which normally forms insulated neighborhoods. If this is true, the author should expect to see the disruption of those insulated neighborhoods in the 104 patients who have matching RNA-seq data. Identification of only several differentially expressed genes was far from evidencing the power of DeepMILO.


Minor,
1.     The model was allele specific. However, the authors did not show data or assessment on how this allelic modeling helped to the performance. As showed in the simulations on Hnisz et al data, the key features were CTCF motif, while most of variation are not hit on the motif. I'm wondering the usefulness of this allele specific modeling.

2.     How they did the test on the Boundary orientation model? As in the test of the boundary model, the AUPRCs were 0.57, 0.747, and 0.841(page 6), while the AUPRC raise to 0.958 (page 7) in boundary orientation model. Isn't the number expected to be lower in boundary orientation model if the boundary model is the base of the former?

3.     From the Method and assessment, I cannot see why DeepMILO can distinguish left and right boundaries of loops that carrying tandem CTCF motifs. Can the authors show how better DeepMILO performed than CTCF-MP or Lollipop on those tandem CTCF appearing boundaries, and comments on why is it the case?

**Reviewer 3**

In this paper the authors developed a deep-learning based method named DeepMILO to predict insulator loops and the effect of genomic variants on their formation. DeepMILO consists of two models: i) a 'boundary model' to predict the regions that can be potential insulator loop anchors and ii) a 'boundary orientation model' to predict left vs right boundaries.
The 'boundary model' is composed of a CNN and an RNN, while the 'boundary orientation model' is composed of two fully connected layers.
The model has some advantages from previous models, however, the authors focused more explaining the performance of the model, but didn't give too much additional insight from their model. It would great if the authors can dig more into the data and provide some additional hints. Below are some of my concerns:


Issues:
1)     As the model is using DNA as input for prediction, how can it be cell-type specific ? Are the users supposed to provide CTCF ChIP-seq peaks as input and get the list of potential loops ? if so, the authors probably should use loops from different datasets and not just the GM12878 for training, or show that GM12878 is sufficient and the addition  another cell-lines does not have a big impact on the prediction accuracy.

2)     Did the authors try different architectures ? if so can they give a summary of the performance of these architectures in the supplementary data to show how they decided on this architecture. For example, does the use of dilated convolutional layers work better than using an LSTM (something similar to David R. Kelley et al, 2019)?

3)     Does Figure 2c show the performance on non-boundary type-1 errors or the global performance ? from the text it seems that it is just about type-1 errors. If so the authors should make that clear in figure legend.
4)     How were the negative boundaries not containing CTCF motif selected ?

5)     Can the authors give more insight from their 'boundary model', for example by exploring the 17bp learned filters (which can be seen as PWMs) learned by the first convolutional layer and see if there any additional motifs that contribute to the prediction ? (similar to for example to Basset, DeepCpG and others). Or by comparing the type of boundaries that the RNN detected by the CNN failed to detect.

6)     For the 'boundary orientation model', the authors should show some representative examples in which the model can successfully predict loops in tandem CTCF motif regions compared to other regions.

7)     It would be interesting to see how does the model predict the effect of genomic mutations on the looping inside CTCF tandem regions.

8)     The authors can go more in details about the structural variations and show which structural variations has the biggest impact on the loop formation. This can be associated with the list of PWMs learned in the first convolutional layer.

Minor issues:
-     In page 3 line 59: Figure 2c is mentioned before figures 2b and 2c.
-     The figure labels should be at the left-side of the figures and not on the center.

**Authors Response**

**Reviewer #1:**
The authors present a novel computational method for estimating the probability of chromatin looping based on DNA sequence, and also for pinpointing mutations (not specific to CTCF motifs) that can alter insulator loops. They point out that their method is similar in its functionality to Lollipop and -mainly- CTCF-MP.
They use 2 types of neural networks, a 2-layer ConvNet and a LSTM-based RNN. First, they train them to predict boundary/anchors (boundary model) and from the surrounding sequence and then boundary orientation (orientation model) from the features they learned during the first part. Finally, their method (DeepMILO) consists of training and ensemble network based on the features learned during the first stage and the predictions of the other two networks to predict the probability that a given pair of sequences form a loop (loop score).
To train their model, they downloaded from ENCODE CTCF/cohesin chromatin loops for the GM12878 and K562 cell lines. These loops were captured by cohesin (RAD21) ChIA-PET and their PET peaks were overlapped with CTCF ChIP-seq peaks. The input to the model is pairs of sequences (4000 for ConvNet, 800 for RNN) and the output loops scores for a predefined set of loops. They split the data into train, validation, and test data-sets.
-- The validation data-set consisted of loops from chromosome 16 of the GM12878 cell line.
-- The testing data-set consisted of chromosomes 7 and 8 from both cell-lines
-- The training data-set consisted of the rest of the chromosomes for the GM12878 cells.
The test set was further divided into categories of different levels of difficulty of detection. 
The authors validated their findings with two experiments.
First, the calculated the loop score changes induced by known loop-disrupting deletions (near TAL1 and LMO2) and noticed significant drops in estimated looping probabilities. They further noticed that main contributors were the 11 positions that matched the CTCFL motif.
Second, they predicted changes in loop probabilities induced by non-coding variants in a set of highly mutated patient samples from ICGC. They noticed that mutational burden is positively correlated (spearman 0.27) with changes in the loop score. Then they found loops that are significantly decreased in 10/241 of the patients and they checked for differential expression between the genes in these loops but they only found 1 out of 14 genes to be DE.
Overall this study start with an interesting idea, but its application is very limited and the models are not tested in a variety of cell lines (possibly due to lack of data). Also, the utility of the model is questionable since when tested in a large patient cohort with high mutational burden, it only produced minimal insights. Perhaps a pan-cancer analysis might have shown more promising results.

***Author response:*** *We would like to thank the reviewer for the comment. We have collected data from more cell lines to train and test our models (discussed in MA.1 above) and run DeepMILO on several ICGC cohorts (MA.2). We also performed an extensive analysis of motifs at loop anchors and found motifs related to binding of RNA (CPEB1) are enriched (discussed in MA.3 above).*

**Major Comments**
-- The authors claim that:
"We developed a powerful method, DeepMILO, for modelling insulator loops and for predicting the effect of variants on these loops. DeepMILO has learned sequence features of insulator loops that are beyond the presence and orientation of CTCF motifs"
This statement seems to imply that DeepMILO can predict non-CTCF related loops despite the fact that it has never seen any non-CTCF related positive example (since the training examples were RAD21 ChIA-PET loops intersected with CTCF ChIP-seq). This reviewer feels that the authors should clarify what is the scope and limitation of their model more adequately. If they believe that their model can consistently identify non-CTCF loops, they should perform some feature extraction analysis, similar to the one they did for their 1 validation experiment, in order to identify which non-CTCF related features are predictive according to their model.

***Author Response****: We thank the reviewer for the comment. We want to clarify that insulator loops require both CTCF and cohesin binding at their anchors. These loops are most relevant and consistent with known knowledge about insulator loops that insulate promoters from enhancers. Insulator loops can be considered as a subset of CTCF loops since not all CTCF loops are insulator loops (many CTCF binding sites do not have cohesin binding). We added a clarification at the beginning of the “Result” section: “Anchors of insulator loops require co-occupancy of both CTCF and cohesin complex (SMC1, SMC3, RAD21 and either STAG2 or STAG1) (Wendt KS et al. Nature 2008), therefore, we only use loops with both CTCF and cohesin at their anchors.”*

CTCF-MP reports much higher AUPRs for the same cell lines. Can the authors explain why they get so different results? It would also be interesting if the authors could extend their analysis to include all 4 cell-lines utilized by CTCF-MP and also test how well their method generalizes across all cell lines.

***Author Response****: Thank you for raising the issue. We note that the datasets used for training and testing the model in CTCF-MP’s paper and our paper are different leading to differential performance of CTCF-MP. CTCF-MP was designed to predict CTCF loops and it was tested to distinguish CTCF loops from non-loops. Our study focuses on insulator loops, which require co-occupancy of CTCF and cohesin at loop anchors. There are other differences in datasets: for example, in CTCF-MP’s test data, loop anchors can have only one CTCF motif, but in our test data, loop anchors can have several CTCF motifs with opposite orientations. These differences have led to the poor performance of CTCF-MP. In this revision, we used CTCF ChIA-PET loops with PET peaks overlapping cohesin (RAD21) ChIP-seq and cohesin (RAD21) ChIA-PET loops with PET peaks overlapping CTCF ChIP-seq from cell lines GM12878, K562, MCF7 and Hela to train and test CTCF-MP and DeepMILO. And performance of CTCF-MP is similar as before. However, we clarified the difference in design goals of CTCF-MP and DeepMILO (in MA.4) and moved the comparison discussion to the supplementary document.*

-- The second validation experiment (patient data) is not very convincing. Only one (BCL2) of the 14 gene seems to be differentially expressed. The reported p-value is high enough to suggest that it could have arisen by chance in 14 tries. More importantly, BCL2 is known to be deregulated by a chromosomal translocation in lymphomas which could potentially induce multiple transcription-regulating events such as the landing position, but the authors only check for CNVs between the groups. More direct evidence, if possible an analysis on paired sequence and ChIP-seq data, is required to establish the clinical relevancy of the method.

***Author response****: We thank the reviewer for the comment. In our revision, we used additional insulator loops from Z. Tang et al. Cell 2015 and thus our model was able to detect changes in more samples. In addition to BCL2, we find that expression of another important oncogene MYC is also associated with loop probability changes. Although we find only two oncogenes with differential expression associated with loop probability changes likely due to limited sample sizes (discussed in MA.2 above), we note that biologically this is a very interesting observation since it points to a likely new mechanism for transcriptional dysregulation of these important genes in lymphoma. Differences in expression of BCL2 and MYC between mutated vs. non-mutated samples are very significant now (P-value ~ 2.77e-9 and 7.49e-5 respectively). We also checked that there are no structural variants involved and there is no difference in copy number of BCL2 and MYC between mutated vs. non-mutated samples.*

*We thank the reviewer for the suggestion of joint analysis of paired whole-genome sequence and ChIP-seq data but unfortunately currently there is no publicly available cohort with a large number of samples with paired CTCF or cohesin ChIP-seq data and whole genome sequencing data for further analysis. We plan to perform this analysis in separate research in the future.*

-- When testing on the K562 cell line, it is important to also test on chromosomes other than chr7 and chr8 to exclude the possibility that these two chromosomes have more easily identifiable loops. Also detailed metrics in each chromosome should be reported to assess the chromosome-to-chromosome variability in performance.

***Author Response****: We thank the reviewer for the comment and suggestion. In this revision, we also tested our models on chromosomes 1 and 14. The result shows that the performance of models is similar when testing on chromosomes 7 and 8, for example, performance of the CNN model on test sets from chromosomes 7 and 8 vs. test sets from chromosomes 1 and 14 is shown in the below table. This result is expected as there is no known knowledge about the differences in chromatin loops between different chromosomes. We hope that the data from chromosomes 1 and 14 can convince the reviewer that it is no easier to identify loops in chromosomes 7 and 8. We did not test all chromosomes due to the large amount of effort needed to train 24 different deep learning models (leave-one-out cross validation) to test each chromosome. In the revised manuscript we mention this in the* ***Data preparation*** *in* ***Methods*** *section: “We checked performance of our models with a test set from chromosomes 1 and 14, and found that performance of models was similar (data not shown), indicating that data from chromosomes 7 and 8 was not easier for models.”.*

Table 1 Performance of the CNN model (in AUPRC) on test sets from different chromosomes

|  | Chromosome 1 and 14 | Chromosome 7 and 8 |
| --- | --- | --- |
| Non-anchor type 1 | 0.882 | 0.866 |
| Non-anchor type 2 | 0.872 | 0.827 |
| Non-anchor type 3 | 0.970 | 0.970 |

-- More importantly, more cell lines should be tested. If no other RAD21 ChIA-PET exists, the authors can be creative and think of other options, e.g. test Hi-C based TAD/sub-TAD boundary loops.

***Author response****: We thank the reviewer for the suggestion. A major change in our revision is that we used data from more cell lines to train and to test our models (MA.1 above). We also used both RAD21 and CTCF ChIA-PET data, which allowed us to have larger datasets for training and testing.*

Minor Comments
-- The color of the nodes in then FC-layer of the neural nets is a bit misleading, it appears as if they are different types of nodes (figures 2b, 3b)

***Author response****: We removed colors of nodes in FC layer in figures 2 and 3.*

-- The authors may want to use the term loop "anchor" instead of "boundary", as "boundary" can easily be confused with TAD boundaries. 

***Author response****: In the revised manuscript we use “anchor” instead of “boundary”.*

**Reviewer #2: ===**
Trieu and Khurana presented a new method to predict the effects of variants on chromatin loops, and therefore possible target genes. The idea of using 3D genome to help decipher variants has been interested in medical research for long time, and a lot of cases have been reported in the literature. However, a tool to systematically address this question remains rare. In that sense, the present work was trying to target an important and interesting question in the field. There are several issues need to be clarified.

***Author response****: We thank the reviewer for acknowledging the importance of the problem and the need for a tool to address it. Below we have clarified the several issues that the reviewer raised.*

Major
1.      The author compared DeepMILO with CTCF-MP. However, the assessment could be misleading.
a)      CTCF-MP was designed and trained by CTCF ChIA-PET data along. In CTCF-MP, a preferred convergent orientation of CTCF is a strong signal for loop prediction. However, in this paper, the authors were using ChIA-PET data of RAD21 to train and test the tools. Moreover, CTCF-MP asked for 520bp should be the flanking of motif center, not the middle of boundaries which were used in this paper. It may unfair to CTCF-MP, although the peak of CTCF and RAD21 are largely overlapped. And overlapping of two datasets could bring more information, per se.

***Author response****: We agree with the reviewer that the comparison is not fair as CTCF-MP was not designed with the same goals as our model. This is discussed under MA.4 above. We acknowledge the difference in design goals between CTCF-MP and DeepMILO and moved the comparison to the supplementary document.*

b)      More important, there are no CTCF motif, or binding, or incorrect orientation in type 2,3,4 and 5. Thus, it is unfair to CTCF-MP in those testing data sets. 
So, why don't use ChIA-PET of CTCF directly?

***Author response****: We agree with the reviewer that the goals of CTCF-MP and DeepMILO are different and as discussed in the comment above we have removed the comparison from the main text. Regarding the use of CTCF ChIA-PET, our study focuses on insulator loops that require both CTCF and cohesin (RAD21) bound at their anchors. These loops can be identified by intersection of cohesin ChIA-PET and CTCF ChIP-seq data or by intersection of CTCF ChIA-PET and cohesin ChIP-seq data. In this revision, we also use CTCF ChIA-PET loops overlapping cohesin (RAD21) ChIP-seq data as pointed by the reviewer to train and test models (discussed in MA.3). CTCF-MP and DeepMILO are given the same training and test data, but the difference in their performances is due to the difference in their design goals. We acknowledge the difference in design goals between CTCF-MP and DeepMILO and this is discussed in the supplementary document.*

2.      The false positive could be over-estimated, as ChIA-PET only captures loops mediated by the targeted proteins, while the predicted loop may not necessarily be mediated by that protein.

***Author response****: We would like to clarify that we predict the changes in loop probabilities for insulator loops mediated by cohesin and CTCF (as captured by ChIA-PET) by mutations. Since we are not predicting loops ab-initio from sequence, we do not expect high numbers of false positives. Additionally, the false positive rate in the revised manuscript is lower since we have used a larger set of insulator loops in this revision (AUPRC shown in Fig 3c).*

3.      The simulations on Hnisz et al data simply means that CTCF motif plays a major role in the loop prediction. Or, in another word, the loops predicted by DeepMILO should largely be CTCF loop, which normally forms insulated neighborhoods. If this is true, the author should expect to see the disruption of those insulated neighborhoods in the 104 patients who have matching RNA-seq data. Identification of only several differentially expressed genes was far from evidencing the power of DeepMILO.

***Author response****: We want to clarify that DeepMILO is designed to learn sequence patterns of insulator loops and predict if mutations can change these sequence patterns. Since not all CTCF motifs are hit by mutations in the 104 patients and the anchors of loops around TAL1 and LMO2 have several CTCF motifs, we do not expect disruption of insulated neighborhood surrounding TAL1 and LMO2 in all 104 patients. Regarding the number of differentially expressed genes, the comment is discussed in detail under MA.2 above.*

Minor,
1.      The model was allele specific. However, the authors did not show data or assessment on how this allelic modeling helped to the performance. As showed in the simulations on Hnisz et al data, the key features were CTCF motif, while most of variation are not hit on the motif. I'm wondering the usefulness of this allele specific modeling.

***Author response****: We compared the performance of models using allele specific information vs. models not using allele specific information and did not see major differences in performance. Moreover, not using allele-specific model allowed us to use more cell lines to train the model since many cell lines have ChIA-PET and ChIP-seq data but only a few also have whole-genome sequencing data. Therefore, in this revision, we removed the use of allele specific information to train the model.*

2.      How they did the test on the Boundary orientation model? As in the test of the boundary model, the AUPRCs were 0.57, 0.747, and 0.841(page 6), while the AUPRC raise to 0.958 (page 7) in boundary orientation model. Isn't the number expected to be lower in boundary orientation model if the boundary model is the base of the former?

***Author response****: We thank the reviewer for the comment. When testing the Boundary (Anchor) model, we used both boundary and non-boundary, but only true boundaries are used to test the Boundary orientation model. Therefore, performance of the Boundary orientation model is independent from performance of the Boundary model. We mention this detail in “Anchor orientation model to distinguish left and right anchors” section: “****DNA sequences of anchors*** *in chromosomes 7 and 8 were used to test the model…”*

3.      From the Method and assessment, I cannot see why DeepMILO can distinguish left and right boundaries of loops that carrying tandem CTCF motifs. Can the authors show how better DeepMILO performed than CTCF-MP or Lollipop on those tandem CTCF appearing boundaries, and comments on why is it the case?

***Author response****: DeepMILO uses not only signal from the Boundary orientation model but also learned features from the Boundary model. Using a threshold that gives a balance between precision and recall in the validation set, 65% of tandem loops in the test set are correctly predicted by the model. In CTCF-MP, the model outputs binary values so that we used a threshold of 0.5 and found that 33.3% of tandem loops are satisfied in the test set.*

*Since DeepMILO was designed to identify loops with anchors bound by both CTCF and cohesin, so we expect it can learn other features besides CTCF related features, while CTCF-MP was designed to identify loops with anchors bound by just CTCF and with motifs in convergent orientation. We think that the difference in design and capability of deep learning vs. capability of the word2vec model have resulted in the difference in performance of DeepMILO and CTCF-MP.*

=========================


**Reviewer #3:** In this paper the authors developed a deep-learning based method named DeepMILO to predict insulator loops and the effect of genomic variants on their formation. DeepMILO consists of two models: i) a 'boundary model' to predict the regions that can be potential insulator loop anchors and ii) a 'boundary orientation model' to predict left vs right boundaries. 
The 'boundary model' is composed of a CNN and an RNN, while the 'boundary orientation model' is composed of two fully connected layers.
The model has some advantages from previous models, however, the authors focused more explaining the performance of the model, but didn't give too much additional insight from their model. It would great if the authors can dig more into the data and provide some additional hints. Below are some of my concerns:

***Author Response****: We thank the reviewer for appreciating the advantages of the model. In the revised version we provide additional insights about the data provided by the model.*

Issues:
1)      As the model is using DNA as input for prediction, how can it be cell-type specific ? Are the users supposed to provide CTCF ChIP-seq peaks as input and get the list of potential loops ? if so, the authors probably should use loops from different datasets and not just the GM12878 for training, or show that GM12878 is sufficient and the addition  another cell-lines does not have a big impact on the prediction accuracy.

***Author response****: We thank the reviewer for the comment. Changes in loops may arise due to epigenetic changes or genome sequence changes. In this work, we focus on loop changes due to sequence variants. Thus, in the strict sense, the model is not designed to predict loop changes due to epigenetic variation across cell-types. In this revision, we used data from four cell lines, GM12878 (B-Lymphocyte), K562 (chronic myelogenous leukemia), MCF7 (breast adenocarcinoma) and Hela (cervix adenocarcinoma) to train and test our models. Thus, the union of insulator loops from these cell types is a more comprehensive list than in the previous version. However, if the user provides loops for their particular cell-type, DeepMILO can predict if sequence changes in patients will disrupt those loops (mentioned in the* ***Loop model for learning features of pairs of DNA sequences of anchors of insulator loops*** *of* ***Results*** *section in the manuscript: “We provide DeepMILO as a software tool to predict the impact…”). With larger training datasets and improved architectures, our models have been improved significantly (figure 2d and 3c), e.g. anchor prediction increases from 0.72 to 0.90 AUPRC.*

2)      Did the authors try different architectures ? if so can they give a summary of the performance of these architectures in the supplementary data to show how they decided on this architecture. For example, does the use of dilated convolutional layers work better than using an LSTM (something similar to David R. Kelley et al, 2019)?

***Author response****: We thank the reviewer for this suggestion. In this revision, we tried dilated convolutional layers and it helped to achieve better performance. In particular, we used three dilated convolutional layers with dilation rates of 1, 3 and 7. And using both dilated convolutional layers and LSTM also led to even better performance for our models. With larger training datasets and improved architectures, our models have improved significantly (figure 2d and 3c), e.g. anchor prediction increases from 0.72 to 0.90 AUPRC.*

3)      Does Figure 2c show the performance on non-boundary type-1 errors or the global performance ? from the text it seems that it is just about type-1 errors. If so the authors should make that clear in figure legend.

***Author response****: The reviewer is correct that it is performance on the non-boundary type 1 test set. We made it clear in the legend.*

4)      How were the negative boundaries not containing CTCF motif selected ?

***Author response****: Non-boundaries without CTCF motifs contain all regions in the genome that do not contain a CTCF motif. We clarified that in “Data preparation” section: “Negative anchor samples without CTCF motifs are all regions of 4,000 bases that are not overlapping with any CTCF motif…”*

5)      Can the authors give more insight from their 'boundary model', for example by exploring the 17bp learned filters (which can be seen as PWMs) learned by the first convolutional layer and see if there any additional motifs that contribute to the prediction ? (similar to for example to Basset, DeepCpG and others). Or by comparing the type of boundaries that the RNN detected by the CNN failed to detect.

***Author response****: We thank the reviewer for this suggestion. In this revision, we implemented the Class Activation Map visualization technique (R. R. Selvaraju et al. 2016) to see what parts of a sequence activated the class of the sequence. We found that many different parts of a sequence of an anchor activate the class of the sequence. Especially, the model appears to focus on regions around the center of the sequence (supplementary figure 1). In contrast, when we looked at a sequence of a non-anchor, there are fewer parts that activated the sequence as an anchor. We added this discussion in “****Motifs at loop anchors****” section. We also looked for motifs in regions where the model focused on during classification (discussed in MA.3 above) and found that the motif CPEB1 related to RNA binding is the most frequent motif. As evidence of RNA being involved in chromatin loops has been shown recently (AS Hansen et al. Molecular Cell 2019), this motif may play a role in regulating chromatin loops. We include this discussion in “****Motif at loop anchors****” section.*

6)      For the 'boundary orientation model', the authors should show some representative examples in which the model can successfully predict loops in tandem CTCF motif regions compared to other regions.

***Author response****: We thank the reviewer for the suggestion. We checked how well tandem loops are satisfied in the test set and find that 65% of them are satisfied. We include below an example of a tandem loop, a divergent loop and a convergent loop sharing an anchor and having loop probability of 0.68, 0.016 and 0.042 respectively. The model correctly predicted loop probability for the tandem loop and the other two non-loops.*

Figure 1 Predicted loop probabilities of a tandem loop and two non-loops with convergent and divergent CTCF motifs predicted

7)      It would be interesting to see how does the model predict the effect of genomic mutations on the looping inside CTCF tandem regions.

***Author response****: We thank the reviewer for the suggestion. There are 8,305 tandem loops in the whole dataset and we noticed that absolute values of loop probability reductions from mutations in patient samples for these tandem loops are comparable with other loops (min, median and max values of loop probability reductions in tandem loops are -0.592, -2.74e-05 and 0.846, comparing with corresponding values from other loops are -0.648, -3.23e-5 and 0.875). This result indicates that genomic mutations have similar effects across tandem and non-tandem loops. We show an example of a tandem loop being disrupted by a mutation below. The mutation hit a CTCF motif at the anchor and changed a C base to A (CTCF motif is* *at chr7:95156918 - 95156936 and the mutation is at chr7: 95156931 - 95156932). The sample is from the Malignant Lymphoma (MALY-DE) cohort.*

Figure 2 A tandem loop affected by a mutation in sample SA596372 of the Malignant Lymphoma (MALY-DE) cohort

8)      The authors can go more in details about the structural variations and show which structural variations has the biggest impact on the loop formation. This can be associated with the list of PWMs learned in the first convolutional layer.

***Author response****: We thank the reviewer for the suggestion. We found that structural variants often have large sizes (larger than 10kb) and cause dramatic changes in loop probability because they often take away entire loops or anchors (anchor median size is 4kb). Thus, their effect on loops are relatively easy to predict as they often reduce loop probability to near zero. For that reason, we focused more on small variants, whose effects are more difficult to predict, in our study. When we implemented the Class Activation Map visualization technique, we found several motifs that activate the class of sequences of anchors. CTCFL is one of the motifs. But the most frequent motif is the RNA binding related motif CPEB1 (more details discussed in MA.3). We included this discussion in “****Motifs at loop anchors****” section.*

Minor issues:
-       In page 3 line 59: Figure 2c is mentioned before figures 2b and 2c.

***Answer****: Thank you for the comment. We fixed the issue.*

-       The figure labels should be at the left-side of the figures and not on the center.

***Answer****: Thank you for the comment. We fixed the issue.*

**Second round of review**

**Reviewer 1**

Major:
This reviewer feels that the authors still don't show convincingly that the model learns anything "beyond the presence and orientation of the CTCF-motifs". In particular, they present 2 types of analyses to interpret their model.

On the one hand, they interpret the prediction of a TAL1 deletion by "inducing artificial mutations" in the anchors under investigation but they find that CTCF is the major driver for the call. This type of analysis seems very revealing and it would be interesting if the authors could extend it to examples from the non-loop type 1 test set in order to demonstrate which non CTCF motifs are picked up by the model.

On the other hand, they use class activation maps (CAM) only for the anchor model. This analysis is not very convincing for the following reasons:
- They only show CAM for a random unspecified anchor. More anchors should be investigated in order to draw conclusions.
- From that they only deduce that the central part of the anchor contributes relatively more but this is not further investigated. It could be because in this part of the sequence has a CTCF motif or because the LSTM model also contributes there.
- Finally, they don't show the full results of motif enrichment analysis just the top 3 motifs.

It is also evident from the precision recall curves that lack of CTCF motifs is a strong predictor of loop disruption so its absence from the top hits raises suspicions about the representativeness of the anchor sample.

The validation with human data is improved in this version of the manuscript. However, the argument that mutational burden appears to be correlated to loop disruption is a little confusing. It seems natural that since they have a model that predicts disruption from mutations more mutations are expected to predict more disruptions. This reviewer feels that further explanation of their rational would benefit the manuscript.

Minor:
- It's not clear why they treated tandem loops differently from the rest in the testing of their model.
- Typo at line 53: "four" is left in by mistake
- Probably typo at line 55: Wilcoxon "signed-rank" test, they probably mean "rank-sum".

**Reviewer 2**

The authors properly addressed all my concerns, particularly, improved the comparison and added new data, which helped the model as well.

**Authors Response**

**Point-by-point responses to the reviewers’ comments:**

**Reviewer #1:**

Major:
This reviewer feels that the authors still don't show convincingly that the model learns anything "beyond the presence and orientation of the CTCF-motifs". In particular, they present 2 types of analyses to interpret their model.

On the one hand, they interpret the prediction of a TAL1 deletion by "inducing artificial mutations" in the anchors under investigation but they find that CTCF is the major driver for the call. This type of analysis seems very revealing and it would be interesting if the authors could extend it to examples from the non-loop type 1 test set in order to demonstrate which non CTCF motifs are picked up by the model.

On the other hand, they use class activation maps (CAM) only for the anchor model. This analysis is not very convincing for the following reasons:
- They only show CAM for a random unspecified anchor. More anchors should be investigated in order to draw conclusions.
- From that they only deduce that the central part of the anchor contributes relatively more but this is not further investigated. It could be because in this part of the sequence has a CTCF motif or because the LSTM model also contributes there.
- Finally, they don't show the full results of motif enrichment analysis just the top 3 motifs.
 
It is also evident from the precision recall curves that lack of CTCF motifs is a strong predictor of loop disruption so its absence from the top hits raises suspicions about the representativeness of the anchor sample.

***Author response:*** *We thank the reviewer for the comment which motivated us to improve the CAM analysis as discussed below. In this revision, we have analyzed more anchors for CAM analysis (in the previous version, the results were reported for the CAM analysis of 100 anchors, although a representative figure was shown for one anchor) and adopted a peak calling approach to help identify transcription factor motifs used by the CNN model more accurately. Additionally, we also want to point out that our model is able to distinguish loop anchors from CTCF ChIP-seq peaks, which shows that it must rely on other features in addition to CTCF motif features. We provide more details for these points as follows.*

- ***High performance of our model to distinguish loop anchors from CTCF ChIP-seq peaks***

*In figure 2d, we show that our model can distinguish loop anchors from CTCF ChIP-seq peaks (non-anchor type 1) with high AUPRC (0.903). Both loop anchors and CTCF ChIP-seq peaks contain CTCF motif, therefore, if our model could not learn anything beyond the presence and orientation of CTCF motif, it could not achieve such a high performance in separating loop anchors and CTCF ChIP-seq peaks.*

***Excerpt from manuscript, “Result - Performance of the anchor model on different test sets” section:***

*“…The first test set contains negative samples from regions containing CTCF motifs overlapping CTCF ChIP-seq peaks but are not anchors of any insulator loop (non-anchor type 1) … The results from the three test sets are shown in Figure 2d. While the model performed well for the non-anchor type 1 test set achieving AUPRC of 0.903 …”*

- ***CAM analysis of 700 anchors shows enrichment of 38 TF motifs including CTCF***

*We would like to clarify that in the original submission, the results reported for the CAM analysis were for 100 anchors, although a representative figure was shown for one anchor. We apologize for not providing all the results of motif analysis. In this revision, we expanded the CAM analysis to use 700 loop anchors. In our original submission, we were looking for continuous regions of high intensity. However, upon manual visualization of CAM output, we find our previous approach did not capture all peak regions, especially regions of mixed bases with high and low intensity. In this revision, we follow a peak calling approach to more accurately capture the trend in the CAM signal output (CAM peak regions). Then we performed TF motif enrichment within those CAM peak regions and found 38 TFs most enriched within these peak regions. We note that some members of the zinc fingers TF family appear significantly enriched and CTCF is ranked 23 in the list. Since our negative set to train the CNN model also contains regions with CTCF, we do not expect to see CTCF in the top of the list. We note that the CAM analysis is restricted to 700 random anchors due to the limited time and time-consuming nature of this analysis.*

***Excerpt from the manuscript, “Result -*** ***Motifs at insulator loop anchors” section:***

*“…For each anchor, we performed peak calling (details in Methods section) to identify regions of high intensity in the CAM signal output (referred to as CAM peaks). On average, there are 29 CAM peaks per anchor. We aggregated relative positions of CAM peaks to anchor centers over the 700 anchors (figure S1) and found that CAM peaks are distributed relatively evenly across the 4,000 bases of anchors. This result shows that the CNN model used many parts of sequences to distinguish anchors from non-anchors. We then used Analysis of Motif Enrichment (MEME suite) [25] to identify known motifs (HOCOMOCO v11 FULL) enriched at CAM peaks (E-value < 0.0001) and found 38 motifs (the list of motifs is shown in table S2). As a key element of insulator anchors, CTCF motif is significantly enriched. In addition, we note that motifs of some other members of the zinc finger TF family (ZN770, ZN121, ZN335, and IKZF1) appear significantly enriched. It is interesting that ZFX is among the enriched motifs. This result is in concordance with the previous analysis in [16]…”*

***Added Table S2: Motifs found by Analysis of Motif Enrichment (AME) from MEME suite in regions used by the CNN model to identify loop anchors***

- ***Location of CAM peak regions***

*We thank the reviewer for the comments about the location of CAM regions. As suggested by the reviewer, we further investigated the location of CAM peak regions for 700 anchors in the CNN model (where the model focused on identifying loop anchors and a part of negative set also contains CTCF motifs) and find that they distribute homogeneously across the 4,000 bases of anchors (Figure 1) while most CTCF motifs occur near the center of anchors (Figure 2). This result demonstrates that the CNN model trained did not just focus on CTCF motifs to identify anchors.*

Figure 1 Number of CAM peaks across anchors

Figure 2 Relative position of CTCF motifs in anchors

- ***Mutations at TAL1 locus***

*Our simulation of artificial point mutations at TAL1 locus demonstrated that in terms of single point mutations at this locus, mutations within CTCF motif have the most dramatic effect on insulator loop probability. We agree with the reviewer that this result is expected. However, it does not rule out the possibility that combinations of different mutations outside of CTCF can also affect insulator loops. We have now clarified this in the revised manuscript.*

***Excerpt from the manuscript, “Result - Validation of DeepMILO with known loop-disrupting deletions” section:***

*“…We find that the DNA sequence of these 11 positions matches well with the CTCF motif (p value = 1.96e-03, Figure S2 c). The reduction in loop probability is as high as 0.70, indicating that small mutations of one base could result in significant impact on insulator loops and that DeepMILO is sensitive to small mutations. These results demonstrate that DeepMILO can be used to identify the exact bases among large deleted sequence whose alteration would be associated with loop disruption. We note that these results were based on individual single-nucleotide mutations and therefore do not rule out the possibility of small mutations of several bases or combinations of small mutations outside CTCF motif causing significant effects on insulator loops.”*

**Reviewer comment:**
The validation with human data is improved in this version of the manuscript. However, the argument that mutational burden appears to be correlated to loop disruption is a little confusing. It seems natural that since they have a model that predicts disruption from mutations more mutations are expected to predict more disruptions. This reviewer feels that further explanation of their rational would benefit the manuscript.

***Author response:*** *We thank the reviewer for the comment. The general trend is that more mutations are associated with more disrupted insulator loops. We now discuss in the manuscript that this likely indicates that, in general, cancers with higher mutational burden also have higher chromatin instability. However, we also note an exception of a group of rare Leiomyosarcoma cancer patients (LMS-FR) with much less mutational burden compared to skin cancer (MELA-AU) but many more disrupted insulator loops. This may indicate a more significant role of chromatin instability in these patients, though further experimental validations will be needed to confirm this.*

***Excerpt from the manuscript, “Result - Identification of disrupted insulator loops in 1,834 patient samples from twelve cancer cohorts” section:***

*“…Mutational burden is strongly correlated with the numbers of disrupted loops (Figure 4 a and b, 0.87 Spearman rank correlation between medians). Thus, in general, more mutations are associated with more disrupted insulator loops. This result indicates that cancers with higher mutational burden are likely to have higher chromatin instability. Leiomyosarcomas (LMS_FR cohort), a type of soft tissue sarcoma, is an exception. It has the most disrupted insulator loops though it has far fewer number of mutations compared to melanoma (MELA_AU cohort) (median number of mutations and disrupted loops are 28,868 and 1,660 in LMS_FR respectively, and 63,571 and 420 respectively in MELA_AU). This may be related to the transcriptional changes associated with tumor evolution in leiomyosarcomas [30]…”*

Minor:
- It's not clear why they treated tandem loops differently from the rest in the testing of their model.

***Author response:*** *We treated tandem loops differently because CTCF motifs in their anchors are not in convergent orientation like most insulator loops. And models relying on the presence and orientation of CTCF motifs alone would not be able to identify this type of loops. The capability of our model to identify these loops further indicates that our model did not rely solely on CTCF motif features to identify insulator loops.*

- Typo at line 53: "four" is left in by mistake

***Author response:*** *Thank you for your comment. We removed the typo.*

- Probably typo at line 55: Wilcoxon "signed-rank" test, they probably mean "rank-sum".

***Author response:*** *Thank for your comment. We meant “rank-sum” test and fixed it in this revision.*


**Reviewer #2**: ===
The authors properly addressed all my concerns, particularly, improved the comparison and added new data, which helped the model as well.

Third round of review

Reviewer 1: No additional comments.
